# Performance, Variance Components, and Acceptability of Pro-vitamin A-Biofortified Sweetpotato in Southern Africa and Implications in Future Breeding

**DOI:** 10.3389/fpls.2021.696738

**Published:** 2021-09-03

**Authors:** Edmore Gasura, Francisca Matsaure, Peter Sekwena Setimela, Joyful Tatenda Rugare, Cacious Stanford Nyakurwa, Maria Andrade

**Affiliations:** ^1^Department of Plant Production Sciences and Technologies, University of Zimbabwe, Harare, Zimbabwe; ^2^Maize Program, International Maize and Wheat Improvement Center, Harare, Zimbabwe; ^3^International Potato Center, Maputo, Mozambique

**Keywords:** orange fleshed sweetpotato, hidden hunger, sub-Saharan Africa, root and tuber crops, orange sweetpotato acceptability

## Abstract

In sub-Saharan Africa (SSA), vitamin A deficiency (VAD) is a major cause of blindness in children under 5 years. Sweetpotato (*Ipomea batatas* L.) is widely grown in this region, and pro-vitamin A varieties could help to combat such problems. Fourteen newly introduced orange-fleshed sweetpotato (OFSP) varieties from the International Potato Centre (CIP) and two local checks were evaluated at four environments using a 4 × 4 triple-lattice design for total tuber yield, marketable yield, unmarketable yield, total tuber numbers, marketable tuber numbers, unmarketable tuber numbers, dry matter content, and sensory characteristics on boiled sweetpotato. Since varieties were previously tested intensively by CIP under diverse conditions, the focus of the current study was to determine their acceptability by farmers. Across-environment ANOVA showed highly significant differences (*P* < 0.001) for environments, genotypes, and genotype × environment interaction (GE) for all traits studied. Variety Cecelia outperformed the rest in three environments. Cecelia, Erica, Ininda, and Lourdes were found to be the top four most stable and high-yielding varieties. Genetic gains of the top four varieties over the preferred local check Mai Chenje ranged from 135 to 184%, and across-environment broad-sense heritability was 60% for tuber yield. Furthermore, farmers accepted the dry matter content (which was >25%) and taste of all the introduced OFSP varieties. Since there was a high acceptability by farmers, introductions from CIP could help improve human nutrition. Despite the appropriate design, the error variance component was the highest for all traits, and proper field plot techniques were proposed in future breeding and testing activities.

## Introduction

Globally, sweetpotato ranks seventh and 13th based on the total production and monetary value, respectively (Ewell and Mutuura, 1991). The crop is a staple diet in some countries in Africa (Low et al., 2009). Over 105 million tons of sweetpotato are produced annually, and Africa accounts for 21% of this production (Mu and Li, 2019). In Zimbabwe, sweetpotato production has been steadily increasing and gaining a wide utilization in face of climate change (Mudombi, 2013; Mwando, 2014). However, low-yielding varieties of poor nutritional quality have been the major challenge in the production in sub-Saharan Africa (SSA) (Jogo et al., 2021). In most African countries, sweetpotato is grown as a supplementary crop, harvested as a piece meal for household consumption and for income generation. Despite various crops being utilized in Africa, vitamin A deficiency (VAD) remains a major public health challenge in developing countries (Burri, 2011; Dube et al., 2014). According to the World Health Organization (WHO), the severity of VAD can lead to disorders, such as xerophthalmia, anemia, and increased susceptibility to infection (Gurmu et al., 2014). Risks associated with VAD disorders are to a great extent raised by low vitamin A (VA) intakes during demanding life situations such as childhood, infancy, pregnancy, and lactation (Niringiye et al., 2014). World development and health agencies have responded to the problem of VAD by distributing VA tablets and fortifying processed food. However, according to Dube et al. (2014) and Kapinga et al. (2003), many resource-limited families in SSA fail to sufficiently and regularly access these supplements.

Orange-fleshed sweetpotato (OFSP) is a cheap and sustainable crop-based source of naturally bioavailable β-carotene (Mekonnen et al., 2015). The human body converts β-carotene to VA *de novo*. In sweetpotato, β-carotene is found in lipid droplets located in the cell protoplasts where it is released during cooking, thereby enhancing bioavailability (Tomlins et al., 2012). A study carried out in South Africa showed that OFSP is efficient in improving VA status of a population (Laurie, 2010). In the study, feeding of primary school children for 53 school days with 125 g of OFSP resulted in improved VA status in terms of liver stores (Laurie, 2010). The development of OFSP has provided the most promising plant source of VA and stands to be an affordable alternative source of VA to the resource-limited rural families (Anderson et al., 2007). In SSA, the HarvestPlus Program has made available more than 30 OFSP genotypes through the introduction and/or evaluation of existing genotypes (Kapinga et al., 2003).

The crop-based approach to combat VAD such as through the use of OFSP is now an international trend (Kapinga et al., 2003). Since the year 2001, 40 partner agencies from nutrition, health, and agricultural sectors have been working together to extend the impact of OFSP in Tanzania, Ghana, South Africa, Mozambique, Uganda, Kenya, and Ethiopia, under the VA for Africa (VITAA) umbrella (Kapinga et al., 2003). According to Low et al. (2009) and Tumwegamire et al. (2005), OFSP now occupies an estimated 5–10% in central Uganda, 10–15% in western Kenya, 1–2% in the lake zone of Tanzania, and 15–20% in southern Mozambique. However, in the rest of SSA, efforts in the development and promotion of β-carotene-biofortified crops were specifically focused on maize, under the steering efforts of the International Maize and Wheat Improvement Center (CIMMYT) with sweetpotato lacking such initiatives. Thus, building on the lessons on pro-VA maize from CIMMYT, a number of OFSP genotypes were introduced from International Potato Center (CIP) Kenya. This was done through collaborative efforts steered by Welthungerhilfe, a German-based organization through its arm on sustainable intensification of market-based agriculture (SIMBA) project funded by the European Union.

When new genotypes are introduced, they face acceptability challenges due to several issues. Acceptability studies done by several researchers found out that the success of any new genotype depends not only on agronomic characteristics but also on its sensory and utilization characteristics (Ssebuliba et al., 2009). Niringiye et al. (2014) reported that the main criteria used by farmers on genotype choice are high yield followed by early maturity, tolerance to diseases, sweetness, low fiber content, and long underground storage. Tumwegamire et al. (2014) pointed out taste as one of the important attributes determining the acceptability of a genotype by farmers. Kapinga et al. (2009) and Abidin et al. (2015) reported that African farmers and consumers are more interested in genotypes that have high yield and dry matter content and are sweet in addition to resistance to weevils and viral diseases. Laurie (2010) pointed out that sweetpotato breeding programs should seriously consider taste as it is the most important trait after the tuber yield.

Tuber yield performance unlike other traits is highly affected by the environment in which the genotype is grown. Thus, the newly introduced OFSP genotypes must be tested for their genotype × environment interaction (GE). The term “GE” refers to the differential response of different cultivars grown in different environments. This differential response is attributable to environmental intrinsic factors such soil characteristics, climatic conditions, and associated pests and diseases. A desirable genotype must produce a high yield in good environments but maintain above-average yield in poor environments, a scenario is referred to as dynamic stability. In a study by Abidin et al. (2015) using the genotype main effect plus genotype × environment interaction (GGE) and additive main effect and multiplicative interaction (AMMI) models, significant effects of GE were found on yield and its components. However, GE effects were less pronounced on quality traits such as dry matter content, protein content, starch, and β-carotene content. Small interactions in quality traits indicate that the selection for such traits could be conducted at fewer environments, even when breeding programs are focused on various agro-ecological regions (Abidin et al., 2015). Selection of appropriate genotypes must be conducted at environments that simulate field conditions of farmers. Thus, an assessment of GE is particularly relevant to countries in SSA, where agro-ecological conditions are very diverse. The aim of this study was to identify farmer-preferred OFSP genotypes with desirable tuber yield and other agronomic traits, and sensory characteristics among the 14 varieties introduced from CIP.

## Materials and Methods

### Description of the Trial Environments

Since the major focus of the current study is on acceptability, a relatively few number of environments were used because these materials were already widely tested by CIP in its testing environments in Kenya and Mozambique, and those sites capture the most farming conditions in SSA. Specifically, the experiment was carried out on-farm in Njelele 1, Njelele 2, and Njelele 3 of Gokwe South District in the Midlands Province of Zimbabwe and on-station at the Department of Crop Science, University of Zimbabwe in Harare Province (Supplementary Table 1). The University of Zimbabwe is located at an altitude of 1,460 m above sea level, latitude 17°50′S and longitude 30°01′E, and receives an average annual rainfall of about 750–1,000 mm. Temperature averages range from 16 to 31°C in summer and 7 to 21°C in winter. The soils are red clays.

Gokwe South District experiences different edaphic and climatic conditions. The southern part receives rainfall ranging from 650 to 800 mm per annum, and the northern area is dryer, receiving between 450 and 650 mm per annum. The district is characterized by generally high temperatures averaging 30–31°C. Rainfall effectiveness is generally reduced due to high temperatures accompanied by moderate rainfall amounts. According to Hanyani-Mlambo et al. (2000), the area experiences severe mid-season droughts, and as such, it is considered marginal for most crop farming activities. Gokwe South District soils are predominantly Kalahari sands. However, the soils differ within specific locations. The southern area of the district has the amorphic order, regosol group soils, which are mainly deep sands with little silt. To the north of the district, the kaolinite order, fersiallitic group soils dominate, and these soils are grayish brown in color, moderately shallow to deep, and formed mainly on sandstones.

### Sweetpotato Varieties

Fourteen OFSP varieties developed by the International Potato Centre (CIP) in Mozambique and Kenya were evaluated against white-fleshed local checks (Mai Chenje and Mukadzi Wanhasi). These two local varieties represent the widely accepted and high-yielding varieties. Furthermore, the two local names have the words “*Mai* and *Mukadzi*,” which means “women,” implying that wise women must grow at least one of them for food security reasons. The new genotypes introduced from CIP were Ininda, Tio Joe, Gloria, Melinda, Erica, Kabode, Emelia, Irene, Cordina, VITAA, Lourdes, Cecilia, Jane, and Sumaia. Germplasm for the 14 genotypes was received from the CIP in April 2015, and the seed was nursed under closed doors to prevent infestation by aphids and whiteflies, which are major vectors of sweetpotato viral diseases.

### Experimental Design

The sweetpotato varieties used in this study were previously extensively tested by CIP and would thus require a few test environments that represent the major sweetpotato production conditions in southern Africa but a thorough understanding of their acceptability by farmers. The trial was laid out in a 4 × 4 triple-lattice design at each environment during the 2015/2016 farming season. A total of 16 genotypes were planted per environment (14 new genotypes and two local checks). The trials were carried out under rain-fed conditions according to the practices of farmers. All activities were done by farmers using a participatory research approach. Before planting, the fields were first ploughed using an ox-drawn plough on the Gokwe environments and using a tractor drawn plough at the on-station environment. Ridges were manually made in the on-station environment. In the on-farm environments, ridges were made using the ox-drawn plough by making two runs on each side of the ridge while the soil was being thrown to the center resulting in a ridge. This resulted in ridges of 0.3 m height, 6 m length, and 0.4 m width. An inter-ridge spacing of 1 m was maintained. Vine cuttings of approximately 0.25–0.30 cm or eight nodes were planted on top of the ridges at a spacing of 0.25 m. Planting was done by burying three nodes in the soil.

A basal dressing with Compound D (7% N: 14% P_2_O_5_: 7% K_2_O) was applied at a rate of 4 g per planting station. Top dressing was done using ammonium nitrate (34.5% N) in two splits at a rate of 3 g per plant per split. The first split was done at 4–8 weeks after planting, and the second split was done at 8–12 weeks depending on the period on which moisture was available. Weeds were mechanically controlled using an ox-drawn plough and by hoe weeding. Frequency of weeding depended on the practice of farmers, and weeding was done as and whenever necessary. The Gokwe farmers have a practice of re-ridging using an ox-drawn plough soon after top dressing. In the process of re-ridging, weeds were controlled.

### Data Collection

The plants were harvested at 4^1/2^ months after planting. The harvesting process was done by a team of farmers using hoes to dig out the tubers. Vines were removed before digging of ridges. After harvesting, total tubers per plot were weighed and graded. Grading was done to separate marketable from unmarketable yield based on tuber size. The tubers were graded as per the practice of farmers using a visual assessment to determine unmarketable tuber sizes. The unmarketable tubers were approximately 20 mm and below in diameter. Dry matter yield for the marketable tubers was determined by chopping healthy, large sweetpotato tubers into pieces and drying them in an oven at 70°C until a constant weight was attained. Dry matter content was expressed as a percentage of fresh weight using the formula: Dry matter% = (dry weight/fresh weight) × 100 (Rukundo et al., 2013).

### Data Analyses

Combined analysis of variance (ANOVA) and genotype plus genotype x environment interaction (GGE) biplot analysis were conducted using Genstat version 14. Combined ANOVA was performed using the following model:

(1) $$\begin{array}{r} {Y_{ij}}_{\left(\text{k}\right)\left(l\right)}=b_{j}\left(r_{k}\right)\left(E_{l}\right)+r_{k}\left(E_{l}\right)+g_{i}+E_{l}+gE_{\left(\text{il}\right)}+{e_{ij}}_{\left(\text{k}\right)\left(l\right)} \end{array}$$

where *Y*_*ij*__(k)__(*l*)_ is the response of the *i*th genotype in the *j*th incomplete block nested within the *k*th replication also nested in the *l*th environment, *b*_*j*_*r*_(k)_*E*_(l)_ is the effect of the *j*th incomplete block nested in the *k*th replication also nested in the *l*th environment (where *j* = 1, 2, 3, 4), *r*_*k*_(*E*_*l*_) is the effect of the *k*th replication nested in the *l*th environment (where *k* = 1, 2, 3), *g*_*i*_ is the effect of the *i*th genotype (where *i* = 1, 2, 3,.16), *E*_*l*_ is the effect of the *l*th environment (where *l* = 1, 2, 3, 4), gE_(il)_ is the interaction effect between the *i*th genotype and the *l*th environment, and *e*_*ij*__(k)__(*l*)_ is the random error term. Variance components were determined by equating mean squares to their respective expectations and solving the equations. Broad-sense heritability (H^2^) (%) values based on genotype means (Hallauer, 2010) for all the traits were calculated as

(2) $$\begin{array}{r} H^{2}=\frac{\sigma_{g}^{2}}{\sigma_{g}^{2}+\frac{\sigma_{ge}^{2}}{e}+\frac{\sigma_{e}^{2}}{re}}\times 100 \end{array}$$

where $\sigma_{g}^{2}$ is the genotypic variance, $\sigma_{ge}^{2}$ is the GE variance, and $\sigma_{e}^{2}$ is the error variance, *r* is the number of replications, and *e* is the number of environments. In a case where trait data were collected in a single environment, the broad-sense heritability was calculated as

(3) $$\begin{array}{r} H^{2}=\frac{\sigma_{g}^{2}}{\sigma_{g}^{2}+\sigma_{e}^{2}}\times 100 \end{array}$$

Model diagnosis was performed using the additive main effects and multiplicative interaction model (Gauch, 2013) based on Gollob's *F*-test (Gollob, 1968). Model diagnostic using the Gollob's *F*-test identified two significant principal components (PCs), thus allowing the AMMI-2 model (equivalent to the GGE2, biplot) to be applied to the data set. The GGE comparison biplot analysis was performed on the adjusted means from across environments using GenStat version 14 software. The model for the GGE biplot used was described by Yan and Kang (2002) as shown below:

(4) $$\begin{array}{r} Y_{ij}-\mu -{\text{β}}_{j}{=}^{k}\sum_{\text{l}=1}{\text{λ}}_{l}{\text{ξ}}_{il}{\text{η}}_{jl}+{\text{ε}}_{ij} \end{array}$$

where *Y*_*ij*_ is the mean yield of the *i*th genotype in the *j*th environment, μ is the grand mean, β_*j*_ is the main effect of the environment *j*, _*l*_ is the singular value of the *l*th PC (*k* = 2 in this case), _*il*_ is the Eigen vector of genotype *i* for PC *l*, _*jl*_ is the Eigen vector of environment *j* for PC *l*, and _*ij*_ is the residual associated with genotype *i* in environment *j*. According to this model, the biplot is based on environment-centered data using GenStat version 14. Visualization of the mean yield and stability of genotypes using a genotype comparison biplot was achieved by representing an average environment with an arrow (Yan and Kang, 2002). In the scatter biplot, the polygon view displaying the which-won-where pattern was formed by connecting the genotype markers furthest away from the biplot origin, such that the polygon contained all other genotypes (Yan and Kang, 2002). The polygon was then dissected by straight lines perpendicular to the polygon sides and running from the biplot origin. Visualization of the mean yield and stability of genotypes using a genotype comparison biplot was achieved by representing an average environment by an arrow. A line passing through the biplot origin to the average environment was drawn followed by a perpendicular line passing through the biplot origin. Relative yield advantage of the best four varieties over the check was calculated as the difference between the mean of each variety and the mean of the check.

### Experimental Design and Procedure for Palatability Evaluations

After the harvesting of the 16 sweetpotato genotypes during the 2015/2016 cropping season, tubers from each genotype were separately boiled. Sensory evaluations, specifically organoleptic tests, were done to assess the taste of boiled tubers. A panel of 9, 26, and 29 farmers at Njelele 1, Njelele 2, and Njelele 3, respectively, was selected as judges to perform the tests on the samples. The central location test (CLT) method was used as the suitable method for the palatability evaluations. The CLT involves a gathering of potential consumers of a product in one central point, at a central homestead of a village in this study (Kiria et al., 2010). The samples were evaluated using a 1–5 hedonic scale similar to the one described by Ahenkora et al. (1999), where 1= very bad and 5 = very good.

Sensory scores were analyzed using nonparametric Kruskal–Wallis H-test using SPSS statistical package version 21. Pairwise comparisons were done using Mann–Whitney U-test with a significant level adjusted to 0.0004 (Bonferroni correction-applied, i.e., 0.05 significant level divided by 120 pairwise comparisons) using SPSS statistical package version 21.

## Results

### Across-Environment ANOVA

Combined ANOVA showed highly significant differences (*P* < 0.001) for environments, genotypes, and GE for all traits studied that include total tuber yield, marketable tuber yield, unmarketable tuber yield, marketable tuber number, unmarketable tuber number, and total tuber numbers (Table 1). There were highly significant differences (*P* < 0.001) in dry matter content (Table 1) among the genotypes. The dry matter percentage for the genotypes ranged from 25 to 42%. The genotype Erica had the least dry matter content of 25%.

**Table 1:** Mean squares from across-environment ANOVA.

**Source**	**Degrees of freedom**	**Total tuber yield**	**Marketable tuber yield**	**Unmarketable tuber yield**	**Marketable tuber number**	**Unmarketable tuber number**	**Total tuber number**	**^†^Dry matter content (%)**
Environments	3	403.755^***^	297.661^***^	8.5859^***^	16488^***^	10199.5^***^	50199^***^	
Environments.replications	8	18.152^***^	14.281^***^	0.2919^***^	290.4^***^	585.2^***^	1601.6^***^	6.516
Environments.replications.blocks	36	15.081^***^	15.234^***^	0.2129^***^	458.8^***^	373.5^***^	1045.8^***^	31.759^***^
Genotypes	15	37.273^***^	38.546^***^	0.9984^***^	714.1^***^	1794.9^***^	3124.6^***^	74.259^***^
Environment*genotype	41	14.607^***^	12.823^***^	0.4967^***^	378.8^***^	633.3^***^	1351.9^***^	
Residual	75	4.18	3.62	0.1518	157.6	235	513.4	3.972
Total	178	18.938	16.467	0.4634	597.6	669.9	1920.5	31.833

*** *Significantly different at 0.001 probability level*;

† *trait measured in one environment*.

### Genotype × Environment Interaction

The additive main effect and multiplicative interaction (AMMI) model managed to split the interaction into two PCs that were significant (*P* < 0.001), while the residual was nonsignificant; hence, the use of AMMI 2 model (two significant PCs) was adequate in explaining the interaction. AMMI 2 model is equivalent to genotype (G) plus genotype × environment (GE) interaction (GGE 2) biplot, which was used in this study. Model fitting using Gollob's F-test (Gollob, 1968) indicated that the first two PCs to be adequate in explaining the two-way data (Table 2) were significant.

**Table 2:** AMMI analysis of variance.

**Source**	**Degrees of freedom**	**Sums of squares**	**Mean squares**	***F***	***P***
Total	191	3,479	18.22	*	*
Treatments	63	2,850	45.24	10.39	0
Genotypes	15	927	61.81	14.2	0
Environments	3	1,246	415.31	22.75	0
Block	8	146	18.26	4.19	0.0002
Interactions	41	677	16.51	3.79	0
IPCA	17	371	21.82	5.01	0
IPCA	15	234	15.58	3.58	0.00005
Residuals	9	72	8.02	1.84	0.06834
Error	111	483	4.35	*	*

### Genotype Comparisons Based on Mean Yield and Stability

**Figure 1:**
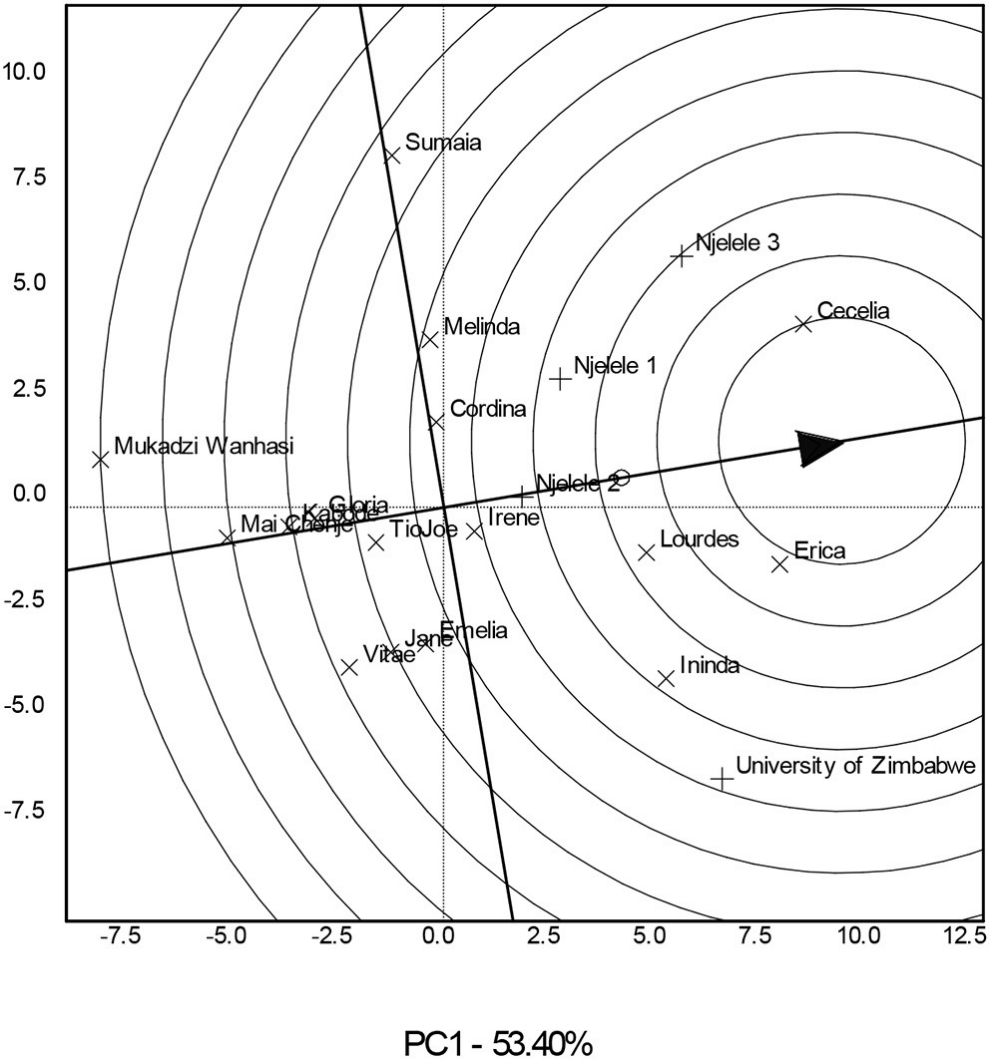
A variety comparison biplot showing the best sweetpotato varieties based on stability and mean performance across four environments.

The GE was significant (*P* < 0.001) (Table 1), and the genotype comparison biplot (Figure 1) indicated that genotypes such as Cecelia, Erica, Lourdes, Ininda, Irene, Cordina, Melinda, and Sumaia had a mean yield greater than that of the grand mean, 9.2 t ha^−1^, for all genotypes (Supplementary Table 2) since they are found in the innermost circles closer to the average environment coordinate represented by an arrow. Genotypes such as Melinda and Sumaia yielded about average but they had poor stability since they are found on the right side of the y-axis but furthest from the arrow. Genotypes such as the check Mai Chenje, Tio Joe, Gloria, and Kabode indicated high stability but are associated with below-average yield since they are found furthest from the average environment coordinate. Genotypes such as Vitae and Jane were neither stable nor high yielding since they are closer to the biplot origin. The top four yielding genotypes were Cecelia, Erica, Lourdes, and Ininda, respectively (Figure 1; Supplementary Table 2).

### Genotypes for Specific Environments

**Figure 2:**
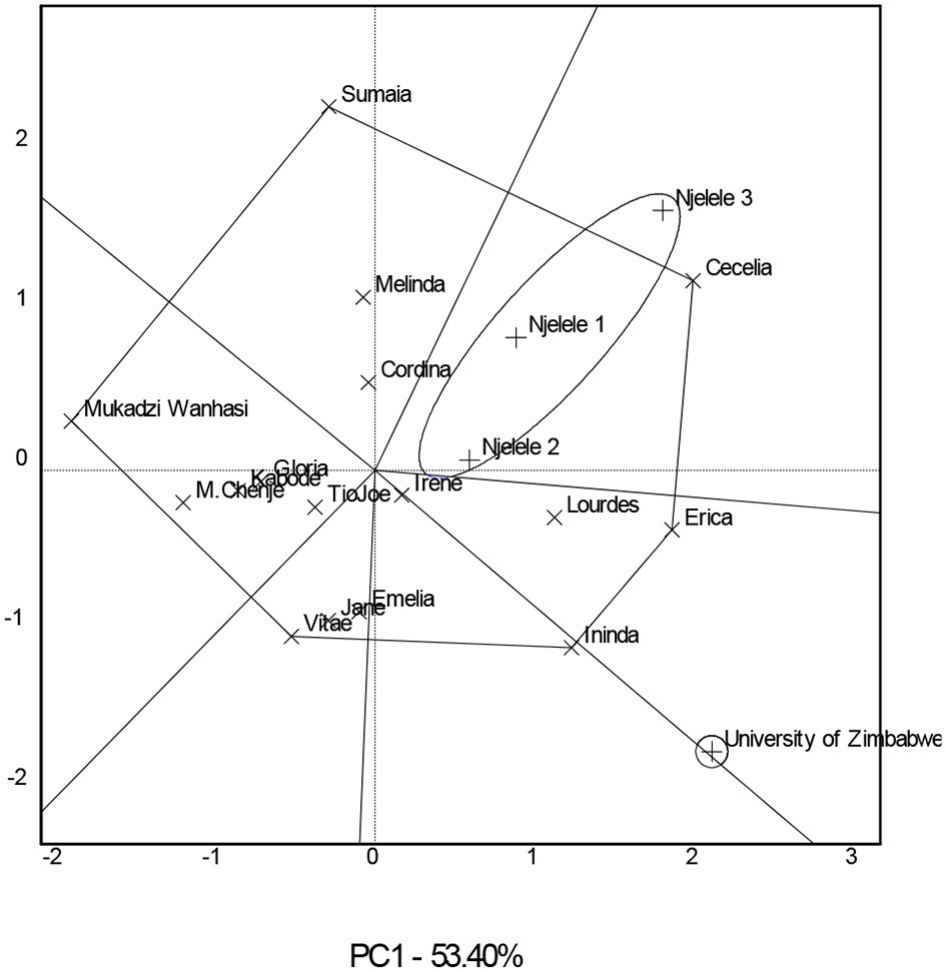
A which-won-where biplot showing mega environments for the 16 sweetpotato genotypes evaluated in four environments.

The significance of crossover GE resulted in the need to identify genotypes that performed best in specific environments. The GGE scatterplot (Figure 2) grouped the three Gokwe South District environments (Njelele 1, 2, and 3) into one mega environment, and the genotype Cecelia outperformed all other genotypes since it is found on the vertex of the polygon in a sector that contains all those environments (Supplementary Table 2). At the University of Zimbabwe environment, genotype Erica was the best since it is found on the vertex of the polygon in a sector that contains this environment. Some experimental genotypes including the checks Mai Chenje and Mukadzi Wanhasi were not specific to any of the environments since they were found in sectors that lacked environments in them. The environments were grouped into two mega environments.

### Variance Components, Heritability Estimates, and Relative Yield Advantage

The variance component due to error was largest at 4.2 followed by variance due to GE (3.5) and lastly variance due to genotypes at 1.9 (Table 3). Across-environment broad-sense heritability was 60% for the total tuber yield. The relative advantage observed for total tuber yield of the better four genotypes, and the best genotype (Cecelia) were 135% and 184%, respectively (Table 3). Interestingly, the relative unmarketable tuber number was negative (Table 3). Mean tuber yield of the best four genotypes (Cecelia, Erica, Ininda, and Lourdes) was 14 t ha^−1^ and was far much greater than the average yield (9.2 t ha^−1^) (Supplementary Table 2).

**Table 3:** Variance components, heritability estimates, and relative advantage of the better varieties over the best check.

**Parameter**	**Total tuber yield**	**Marketable tuber yield**	**Unmarketable tuber yield**	**Marketable tuber number**	**Unmarketable tuber number**
Variance of genotypes	1.9	2.14	0.04	27.9	96.8
Variance of genotype × environment	3.5	3.07	0.11	73.7	132.8
Variance error	4.2	3.62	0.15	157.6	235.0
Heritability (%) across environments	0.6	0.67	0.50	0.5	0.6
Mean of best four varieties	8.3	7.62	0.70	40.4	22.0
Advantage of the best four over the best check (Mai Chenje) (%)	135.1	156.61	22.64	120.1	−5.8
Advantage of best variety (Cecelia) over the best check (%)	184.1	218.93	1.76	134.9	−35.1

### Palatability Tests

Significant differences (Kruskal–Wallis H-test: *P* < 0.001) were recorded among the 16 genotypes in terms of taste (Table 4). Erica was significantly different from Kabode and Vitae only (Mann–Whitney U-test: *P* < 0.0004 with Bonferroni correction applied) in terms of taste. Also, significant differences were recorded between Jane and Kabode (Mann–Whitney U-test: *P* < 0.0004 with Bonferroni correction applied) (Table 4).

**Table 4:** Taste mean ranks and median scores of farmers across Njelele 1, Njelele 2, and Njelele 3.

**Genotype**	**Sample size**	**Mean rank**	**Median score**
Jane	64	573.06^ac^^†^	3
Kabode	64	404.88^b^	1
Irene	64	442.84^abc^	1
Erica	64	584.13^c^	3
Gloria	64	475.52^abc^	2
Cordina	64	430.95^abc^	1
Mukadzi Wanhasi	64	508.98^abc^	2
Summai	38	532.49^abc^	3
Lourdes	64	499.8^abc^	2
Cecilia	64	462.55^abc^	2
Vitae	64	407.49^ab^	1
Mai Chenje	64	482.98^abc^	2
Ininda	64	462.2^abc^	1.5
Emilia	64	534.73^abc^	3
Tio Joe	64	472.45^abc^	2
Melinda	38	555.78^abc^	3
Total	972		
Kruskal Wallis H (Chi-square) value	39.678		
Degrees of Freedom	15		
*p*	0.001		

† *Figures followed by the same letter are not significantly different after subjected to Mann–Whitney U-test: P < 0.0004 (with Bonferroni correction-applied) pairwise comparisons*.

## Discussion

### Genotype × Environment Interaction and Variance Components

The significant differences between the environments are a result of inherent differences in the environmental conditions and reflect that the study environments were diverse. Setimela et al. (2005) divided production environments into various agro-ecological regions based on agricultural production potential, and these agro-ecological zones vary in soil characteristics, rainfall, and temperatures leading to a significant GE. The large error variance components and GE obtained in this study pose challenges in the selection of adapted genotypes and in breeding as they hinder repeatability of the study, thereby stalling efforts in breeding and selection of new genotypes (Gasura et al., 2015). Kamutando et al. (2013) highlighted that large GE and error variance components increase the cost of evaluation as there is a need to increase the numbers of replications, locations, and even years to improve heritability and consequently selection efficiency. The significant GE accompanied by its variance component that more than doubled the variance component for genotypes raises the need of identifying approaches to deal with it. Bernardo (2002) highlighted three options of dealing with significant GE, and these include exploiting or avoiding it. When exploiting GE, breeders identify the stable and high-yielding genotypes, while when avoiding GE, breeders would stratify environments into more uniform mega-environments in which the extent of GE would be minimal. The later approach is not feasible in this study because the data were from a single season. Yan and Tinker (2005) highlighted that the which-won-where pattern and mega-environment delineation are valid if they are repeatable over seasons or years. However, given that the single-season data were present in this case, identifying the stable and high-yielding genotypes was the viable option.

### Genotype Comparisons Based on Tuber Yield and Stability

Tuber yield is the most important trait as it is the one giving an economic benefit to the farmers and the consumers. Good sweetpotato genotypes should produce high yields and should remain stable across varying environments. According to Yan and Tinker (2005), GE interactions are the major causes of the differences in genotypes on their yield stability. Genotypes such as Cecelia, Erica, Ininda, and Lourdes were high yielding, and they demonstrated above-average stability and are desirable. These can be further evaluated for genotype × year interactions. Some genotypes such as Mai Chenje, Gloria, and Tio Joe gave considerable stability but had below-average yields and are not desirable because they produce uneconomic yields when the farmer wants to get higher returns per dollar invested (Kamutando et al., 2013). As a result, plant breeders select higher-yielding genotypes and discard those that are low-yielding regardless of their stability across different environments. Genotypes with low yields and below-average stability such as Vitae, Jane, and Mukadzi Wanhasi are considered poor, and as a result, they have to be discarded as they offer little benefits to the farmer. The observed sweetpotato yields are in line with the average yield of 6 t ha^−1^ reported in various parts of Africa. However, there is potential to get much higher: For example, in southern Africa, the yields of 25 t ha^−1^ were reported, while an average of 14 t ha^−1^ and a yield potential of 18 t ha^−1^ were reported worldwide (Chagonda et al., 2014).

### Genotype and Associated Trait Analysis

Interestingly, the first four high-yielding and stable genotypes were also associated with other desirable traits (results not shown). The high values of heritability (> 50%) for most traits (Table 3) qualify the genotype × trait scatterplot to be more appropriate to examine the correlation between genotypes and the traits across environments (Yan and Kang, 2002). The best four genotypes, Cecelia, Erica, Ininda, and Lourdes, have desirable traits, which include high marketable tuber numbers and tuber yield. Genotypes such as Cordina, Emelia, Tio Joe, and Irene are correlated with undesirable traits in the form of high unmarketable tuber numbers and high unmarketable yield and are highly undesirable. These genotypes are also associated with high total tuber numbers; however, the tuber numbers were high but with a large proportion of unmarketable tubers. It is apparent that these genotypes are very prolific but they fail to support enough tuber expansion. Mai Chenje, Gloria, Vitae, and Kabode were not associated with any of the traits under consideration. This means that these genotypes posed both the desirable and the undesirable traits and thus are not ideal genotypes for the farmers. Dry matter for all the genotypes under study was above 25%, and this is an indication of high dry matter. According to Tumwegamire et al. (2014), a dry matter content of 25% is acceptable to most African consumers. The dry matter content of most of the genotypes tallied with what is expected in good genotypes as documented in the CIP variety catalogue of 2014.

### Farmer Preferences on Genotypes

For farmers to adopt new genotypes, the genotypes must satisfy the expectations of farmers in terms of yield, disease resistance, taste, texture, flavor, and cooking quality among other traits (Ssebuliba et al., 2006). In this study, all the genotypes of farmers were on the extremely low-yielding side, ranking from 13th to 16th positions out of the 16 genotypes evaluated. This could be partly due to the fact that genotypes of farmers could be having latent infections of the viruses that may cause yield reduction (Mukasa et al., 2006; Gasura and Mukasa, 2010). Farmers rated all the introduced genotypes as high yielding. Farmers also considered various traits in genotype selection such as skin color, flesh color, and dry matter content-to-sugar content ratio (as rated by taste that must not be too sweet or too flat) in addition to yield, resistance to weevils (*Cylas spp)* and *sweetpotato virus disease* (SPVD) caused by a dual infection of *Sweetpotato chlorotic stunt crinivirus* (SPCSV) and *Sweetpotato feathery mottle ipomovirus* (SPFMV) as well as *Alternaria* stem and leaf spot disease. SPVD has a wide distribution and is considered as a serious problem in Africa. SPVD can result in 56–98% yield reduction (Ndunguru and Kapinga, 2007), SPFMV strains result in only mild initial symptoms in many cultivars, and they usually recover and may contain low virus titers. Co-infection between SPFMV and SPCSV somehow interferes with recovery and usually causes severe SPVD even in resistant cultivars.

However, in addition to high tuber yield, the OFSP genotypes evaluated possessed various attributes that include varied tuber shape, color, and size. The tuber shapes ranged from thick rounded to long and cylindrical, while skin color ranged from white, cream, to red. The flesh color also varied ranging from deep orange to light orange. The occurrence of varying attributes in the evaluated sweetpotato enables the farmers to identify a combination of choice. High dry matter is the major characteristic used by sweetpotato processors and consumers in SSA (Mwanga et al., 2007). According to Shumbusha et al. (2010), dry matter content is affected by a number of factors, which include genotype, cultural practices, location, climate, soil types, and incidence of pests and diseases. However, in this study, all genotypes had dry matter content above 25% as required. Dry matter content above 25% is an important factor for the preference of a new genotype by consumers (Shumbusha et al., 2010). Furthermore, all the genotypes had a dry matter content above 25% and were all rated as good as the white counterparts. The existence of various traits in sweetpotato offers an excellent opportunity to accommodate various uses (Tumwegamire et al., 2014).

In addition, palatability tests revealed that OFSP genotypes such as Kabode, Irene, Viate, Cordina, and Ininda were ranked best by farmers in terms of taste ahead of the two local checks Mai Chenje and Mukadzi Wanhasi. Generally, it was observed that all the OFSP genotypes had a desirable taste to consumers, implying that these new genotypes were acceptable to the community.

### Potential Adoption of Orange Sweetpotato and Its Expected Impact

The sweetpotato genotypes that are currently being produced in most countries in SSA are mostly white-fleshed and have low nutritional quality when compared to OFSP (Burri, 2011). The production of white-fleshed, low-quality genotypes used to be mainly due to the unavailability of high-quality OFSP genotypes, making these genotypes uncommon in sweetpotato-growing communities where they are needed most. However, VAD is a public health problem in SSA (Dube et al., 2014), leading to blindness in children under the age of 5 years. According to a survey carried out by Dube et al. (2014), up to 90% of children in southern Africa could not receive VA supplementation under the national VA supplementation program. In view of this shortfall in supplementing VA, the production of crops rich in VA such as OFSP genotypes is a good alternative (Burri, 2011). Sweetpotato requires low farming inputs, has high productivity per unit area, has good nutritional value, and is a common crop in most rural areas (Koala et al., 2013). As a result, there is a potential for the reduction of VAD through the replacement of white-fleshed sweetpotato genotypes with OFSP genotypes, which have higher quantities of β*-*carotene if the genotypes are adopted by farmers (Burri, 2011). OFSP contributes to higher VA than other vegetables with β-carotene. In a study carried out by Tomlins et al. (2007), dark OFSP contributed to equal VA to that contributed by carrot genotypes and much higher VA than pumpkin, butternut, and Swiss chard (Laurie, 2010). This confirms the value by which OFSP can make an impact in dietary improvement programs to address VAD. OFSP varieties have high β-carotene levels and can be used as a means to alleviate VAD in low-income communities (Carey et al., 1998). Production and consumption of OFSP genotypes will result in diversity in household food and also nutrition, leading to the alleviation of VAD and hunger. OFSP production will also offer important benefits to people affected by VAD in the rural areas, where, according to Kapinga and Carey (2003), conventional means for curbing VAD such as the supplementation and fortification of foods are less efficient due to infrastructural deficits common in most developing countries.

In addition, a diverse range of products made from OFSP will significantly result in increased farmer income through small businesses and in the process contribute to the improvement of standards of living of the poor rural families. Income generated from selling OFSP products such as vines, tubers, flour, and baked products will help farmers meet family needs such as paying school fees, building better houses, and meeting medical expenses among other needs. Sweetpotato is an already widely grown crop as a secondary food crop throughout almost all of SSA; therefore, promoting a shift in dietary practices, such as changing genotypes, is likely to be easier than introducing a completely new food into the diet (Tomlins et al., 2007). According to Burri (2011), the impact of OFSP genotypes replacing white-fleshed genotypes is great. As reported by Low, 2010, a great proportion of the population at risk of VAD in countries with high sweetpotato production density such as Burundi, Rwanda, and Uganda has fully benefited from the replacement of white-fleshed genotypes with OFSP genotypes.

## Conclusions

OFSP genotypes performed better than the local checks in terms of yield and related traits. All genotypes had acceptable dry matter content (> 25%). Stable and high-yielding genotypes are in the order Cecelia >Erica > Lourdes, and Ininda, and the relative yield advantages were above 100%, ranging from 135 to 184% if the best four genotypes are grown. Farmers preferred the OFSP genotypes due to their high yield and good taste. The results demonstrated the feasibility of adoption of OFSP as an option to ameliorate VAD in SSA.

## Data Availability Statement

The raw data supporting the conclusions of this article will be made available by the authors, without undue reservation.

## Author Contributions

All authors listed have made a substantial, direct and intellectual contribution to the work, and approved it for publication.

## Conflict of Interest

The authors declare that the research was conducted in the absence of any commercial or financial relationships that could be construed as a potential conflict of interest.

## Publisher's Note

All claims expressed in this article are solely those of the authors and do not necessarily represent those of their affiliated organizations, or those of the publisher, the editors and the reviewers. Any product that may be evaluated in this article, or claim that may be made by its manufacturer, is not guaranteed or endorsed by the publisher.
