# Supplementary material for: Performance, Variance Components, and Acceptability of Pro-vitamin A-Biofortified Sweetpotato in Southern Africa and Implications in Future Breeding
Source: Front Plant Sci. 2021 Sep 3;12:696738. doi: 10.3389/fpls.2021.696738 (PMC8446612; doi:10.3389/fpls.2021.696738)
Supplement: Supplementary file 1 [file Table_1.DOCX]

Table S1. Description of the test environments used in the study

| **Environment** | **Altitude (m)** | **Latitude** | **Longitude** | **Rainfall 2015/16** | **Temperature** | **Soil type** |
| --- | --- | --- | --- | --- | --- | --- |
| Njelele 1 | 929 | -18.2544 | 29.2611 | 594.3 | 36°C | Heavy black clay |
|  |  |  |  |  |  |  |
| Njelele 2 | 1226 | -18.3217 | 29.1091 | 291.5 | 31°C | Heavy red clay |
|  |  |  |  |  |  |  |
|  |  |  |  |  |  |  |
| Njelele 3 | 1112.2 | -18.3928 | 28.5604 | 380.5 | 31°C | Sandy- loam |
|  |  |  |  |  |  |  |
| University of Zimbabwe | 1 460 | 17.7824 | 31.0546 | 851.2 | 29°C | Heavy Red clay |
