# Supplementary material for: Performance, Variance Components, and Acceptability of Pro-vitamin A-Biofortified Sweetpotato in Southern Africa and Implications in Future Breeding
Source: Front Plant Sci. 2021 Sep 3;12:696738. doi: 10.3389/fpls.2021.696738 (PMC8446612; doi:10.3389/fpls.2021.696738)
Supplement: Supplementary file 2 [file Table_2.DOCX]

**Table S2. Genotype trait means across the four environments used**

| Variety | Yield t/ha | TY | MTY | UMTY | MTN | UMTN | TTN | DM% |
| --- | --- | --- | --- | --- | --- | --- | --- | --- |
| Ininda | **11.8** | 7.072 | 6.408 | 0.6645 | 44 | 20.96 | 64.96 | 36.86 |
| Erica | **14.6** | 8.768 | 8.109 | 0.6594 | 36.58 | 21.5 | 58.08 | 24.98 |
| Irene | **10.1** | 6.032 | 5.043 | 0.9885 | 37.68 | 40.5 | 78.18 | 37.95 |
| Gloria | **5.9** | 3.53 | 2.464 | 1.0659 | 25.12 | 38.22 | 63.33 | 37.07 |
| Kabode | **5.7** | 3.392 | 2.86 | 0.5315 | 14.63 | 15.02 | 29.65 | 28.77 |
| Emelia | **7.4** | 4.421 | 3.141 | 1.2802 | 26.92 | 43.43 | 70.36 | 35.96 |
| Cecelia | **16.7** | 10.047 | 9.469 | 0.5781 | 43.08 | 15.17 | 58.25 | 28.35 |
| Cordina | **9.4** | 5.629 | 3.999 | 1.6299 | 36.92 | 65.71 | 102.64 | 32.55 |
| Lourdes | **12.3** | 7.374 | 6.489 | 0.8849 | 37.83 | 30.5 | 68.33 | 29.27 |
| Jane | **6.9** | 4.122 | 3.259 | 0.8632 | 23.38 | 31.38 | 54.76 | 31.79 |
| Tio Joe | **7.1** | 4.279 | 3.2 | 1.0787 | 30.47 | 43.05 | 73.52 | 26.64 |
| Vitae | **6.1** | 3.65 | 3.04 | 0.61 | 19.62 | 16.67 | 36.29 | 34.38 |
| Mai Chenje | **5.9** | 3.537 | 2.969 | 0.5681 | 18.34 | 23.38 | 41.72 | 42.37 |
| Grand mean | **9.2** | 5.527154 | 4.65 | 0.877146 | 30.35154 | 31.19154 | 61.54385 | 32.84154 |

TY – total yield per plot, MTY – marketable tuber yield per plot, UMTN – unmarketable tuber number per plot, UMTY – unmarketable tuber yield per plot, TTN – total tuber number per plot, DM%- Percentage dry matter content.
